# *Variovorax* sp. strain P1R9 applied individually or as part of bacterial consortia enhances wheat germination under salt stress conditions

**DOI:** 10.1038/s41598-024-52535-0

**Published:** 2024-01-24

**Authors:** Jacquelinne J. Acuña, Joaquin I. Rilling, Nitza G. Inostroza, Qian Zhang, Lukas Y. Wick, Angela Sessitsch, Milko A. Jorquera

**Affiliations:** 1 Laboratorio de Ecología Microbiana Aplicada (EMALAB), Departamento de Ciencias Químicas y Recursos Naturales, Universidad de La Frontera, Ave. Francisco Salazar 01145, Temuco, Chile; 2 Network for Extreme Environment Research (NEXER), Scientific and Technological Bioresource Nucleus (BIOREN), Universidad de La Frontera, Ave. Francisco Salazar 01145, Temuco, Chile; 3 Millennium Institute Center for Genome Regulation (MI-CGR), Valenzuela Puelma 10207, 7800003 La Reina, Chile; 4 College of the Environment and Ecology, Xiamen University, Xiamen, 361102 China; 5 Fujian Provincial Key Laboratory for Coastal Ecology and Environmental Studies, Xiamen University, Xiamen, 361102 China; 6 Department of Applied Microbial Ecology, Helmholtz Centre for Environmental Research-UFZ, Permoserstraβe 15, 04318 Leipzig, Germany; 7 Bioresources Unit, AIT Austrian Institute of Technology, Konrad-Lorenz-Straße 24, 3430 Tulln, Austria

**Keywords:** Applied microbiology, Abiotic

## Abstract

Endophytes isolated from extremophile plants are interesting microbes for improving the stress tolerance of agricultural plants. Here, we isolated and characterized endophytic bacteria showing plant growth-promoting (PGP) traits from plants in two extreme Chilean biomes (Atacama Desert and Chilean Patagonia). Forty-two isolates were characterized as both halotolerant auxin producers (2–51 mg L^−1^) and 1-aminocyclopropane-1-carboxylate (ACC)-degrading bacteria (15–28 µmol αKB mg protein^−1^ h^−1^). The most efficient isolates were tested as single strains, in dual and triple consortia, or in combination with previously reported PGP rhizobacteria (*Klebsiell*a sp. 27IJA and 8LJA) for their impact on the germination of salt-exposed (0.15 M and 0.25 M NaCl) wheat seeds. Interestingly, strain P1R9, identified as *Variovorax* sp., enhanced wheat germination under salt stress conditions when applied individually or as part of bacterial consortia. Under salt stress, plants inoculated with dual consortia containing the strain *Variovorax* sp. P1R9 showed higher biomass (41%) and reduced lipid peroxidation (33–56%) than uninoculated plants. Although the underlying mechanisms remain elusive, our data suggest that the application of *Variovorax* sp. P1R9, alone or as a member of PGP consortia, may improve the salt stress tolerance of wheat plants.

## Introduction

All plants are inhabited by diverse microbial communities that play crucial roles in plant development, growth, fitness, and diversification. Microorganisms colonizing internal plant tissues for at least part of their lifetime are termed endophytes^1^. Plants growing in extreme ecosystems such as deserts or semiarid areas often harbor a vastly undiscovered diversity of bacterial endophytes, which are well adapted to their hosts and local soil and environmental conditions. Since many bacterial endophytes show plant growth-promoting (PGP) effects^2,3^, endophytes of plants growing under extreme conditions may play a crucial role in the adaptation of plants to nutrient-poor or salt-stressed soils^4^.

Plant growth-promoting bacteria (PGPB), including endophytes, employ different mechanisms to support plant growth, plant health and stress resilience. Endophytic bacteria, similar to bacteria colonizing the plant rhizosphere, may stimulate plant growth via hormonal effects (e.g., auxin production)^5^ or support nutrient acquisition by phosphorus mobilization^6^ and nitrogen fixation^7^. Endophytes may also suppress diseases, e.g., by niche occupation, out-competition, or producing antagonistic effects against pathogens^8^. Plant stress tolerance may be enhanced by several mechanisms^9–11^, including the production of aminocyclopropane-1-carboxylic acid (ACC) deaminase^12^, lowering ethylene levels produced under stress conditions or fostering protective plant antioxidant mechanisms^5^.

Due to long-term selection and coevolution of their bacterial communities, plants thriving in extreme habitats may represent an untapped source for biotechnologically relevant bacteria^13^. Previous studies have showed that the inoculation of rhizosphere bacteria from native plants grown in extreme environments (Atacama Desert and the Chilean Andes) increased the growth and stress tolerance of wheat plants exposed to salinity and water shortage conditions^14,15^. However, to date, mostly endophytes of crops have been investigated regarding their function in and importance for plant growth under stress conditions.

In agriculture, PGPB have been primarily applied as inoculants consisting of individual strains. However, there are increasing attempts to develop tailored bacterial consortia to harness synergetic beneficial effects not obtained by using single species only^16^, although the mechanisms leading to such synergetic PGP effects remain vastly elusive. For instance, several studies have demonstrated that two- or three-member consortia enable beneficial plant physiological and biochemical responses to abiotic stress^14,15,17,18^. In another study, Sun et al.^19^ described synergistic activities between *Bacillus velezensis* SQR9 and an endophytic *Pseudomonas stutzeri* strain leading to increased plant growth and salt stress tolerance of cucumber plant seedlings. Therefore, bacterial interactions contribute to the effectiveness of bacterial consortia.

Here, we hypothesized that endophytic bacteria associated with extreme Chilean biomes (Atacama Desert and Patagonian) can be applied individually or as bacterial consortia to improve the germination of seeds and growth of wheat plants exposed to varying salt stress conditions (0.15 M and 0.25 M NaCl) under greenhouse and field conditions. In this study, wheat was chosen because it is highly relevant for Chilean agriculture, which is being significantly impacted by climatic events^20,21^, including higher temperatures and droughts, with concomitant lower moisture and higher concentrations of salts in soils.

## Results

### Isolation and characterization of putative PGP endophytes

In total, 376 strains were obtained from root and leaf tissues of plants growing in the Atacama Desert (*Distichlis spicata* and *Pluchea absinthoides*) and Chilean Patagonia (*Gaultheria mucronata* and *Hieracium pilosella*) (Table 1). Of all isolates, 18% (68 isolates) were able to grow on media with restricted P (bacterial P mineralization and/or solubilization; Table 1). Among the 68 preselected P-utilizing isolates, 64 (94%) were able to grow on DF-minimal medium supplemented with ACC as the sole N source and to produce tryptophan-dependent auxins. ERIC–PCR thereby revealed 42 nonredundant isolates (Table 1). They all showed ACC deaminase activity (4.7 to 27.7 µM α-ketobutyric acid mg^−1^ h^−1^), while 35 of the 68 preselected P-utilizing isolates (84%) produced tryptophan-induced auxins (0.1–52.1 mg L^−1^; Table 2). However, IAA synthesis (0.2–1.3 mg L^−1^) was solely found in strains P1H14, ER1B, NR3B, and NR1A (Table 2). Thirty-three (79%) of the 42 isolates were halotolerant (≥ 5% NaCl). All isolates from *D. spicata* (Atacama Desert) showed high halotolerance (10% NaCl), while only three isolates from *P. absinthoides* (Atacama Desert) were halotolerant (strains P2H47 and P2R32 and P2R43). All isolates from Patagonian *G. mucronata* were moderately halotolerant (5 and 7.5%), with only strain NH1B being highly halotolerant (10% NaCl). Except for strain ER1B, all isolates from *H. pilosella* were halotolerant at 10% NaCl (Table 2).

**Table 1:** Number of isolates on LB and NM-1 minimal medium during sequential selection of putative plant growth-promoting (PGP) bacteria from plants in the Atacama Desert (*Distichlis spicata* and *Pluchea absinthoides*) and Chilean Patagonia (*Gaultheria mucronata* and *Hieracium pilosella*).

Selection for P solubilization					Sub-total^a^	Selection for N utilization and phytohormone production		Selection for genotype
Plant species		NBRIP	PVK	PSM		DF-ACC	Auxins	ERIC-PCR
Atacama Desert								
*D. spicata*	166	36	31	72	27	26	26	17
*P. absinthioides*	92	29	27	33	17	14	14	12
Chilean Patagonia								
*G. mucronata*	53	14	22	12	9	9	9	8
*H. pilosella*	65	41	46	21	15	15	15	5
Total	376	120	126	138	68	64	64	42

PGP is based on the detection of representative plant growth-promoting traits.

*ERIC-PCR* genotyping of strains by enterobacterial repetitive intergenic consensus, *NBRIP* National Botanical Research Institute’s phosphate growth medium supplemented with insoluble (inorganic) tricalcium phosphate as sole P source, *PVK* Pikovskaya’s medium supplemented with insoluble tricalcium phosphate as sole P source, *PSM* phytate-screening medium supplemented with insoluble (organic) phytic acid dodecasodium salt hydrate as the sole phosphorus source, *DF-ACC* growth in DF medium supplemented with 1-aminocyclopropane-1-carboxylic acid (ACC) as the sole source of nitrogen and carbon, *auxin* production of tryptophan-dependent auxins revealed in Luria–Bertani medium by Salkowski reagent.

^a^Isolates showing inorganic and organic phosphate utilization.

**Table 2:** Quantification of ACC deaminase activity and production of tryptophan-dependent auxins in PGP endophytic bacterial isolates from plants in the Atacama Desert (*D. spicata* and *P. absinthoides*) and Chilean Patagonia (*G. mucronata* and *H. pilosella*).

Plant species	Isolate	ACC deaminase activity (µM α-ketobutyric acid mg^−1^ h^−1^)	Production of tryptophan-dependent auxins (mg L^−1^)		Salt tolerance (% NaCl)
			Total	IAA	
*D. spicata*	P1H14	11.7 ± 1.3^†^	40.4^‡^	1.3	10
	P1H28	10.8 ± 1.7	38.5	N.D.	10
	P1H34	10.4 ± 1.4	0.0	N.D.	10
	P1R2	8.6 ± 0.5	3.8	N.D.	10
	P1R9*	**19.3 ± 3.4**	**41.1**	**N.D.**	**10**
	**P1R11**	**25.4 ± 5.1**	**35.9**	**N.D.**	**10**
	**P1R13**	**16.4 ± 0.6**	**38.8**	**N.D.**	**10**
	P1R15	8.0 ± 0.6	44.4	N.D.	10
	P1R16	15.4 ± 8.3	41.5	N.D.	10
	P1R29	7.0 ± 1.2	6.5	N.D.	10
	**P1R34**	**23.6 ± 7.7**	**42.4**	**N.D.**	**10**
	P1R39	16.1 ± 4.9	38.6	N.D.	10
	P1R41	19.0 ± 2.9	5.08	N.D.	10
	P1R42	13.4 ± 2.6	43.0	N.D.	10
	P1R65	11.3 ± 5.7	7.67	N.D.	10
	P1R73	7.6 ± 0.7	48.1	N.D.	10
	P1R81	9.5 ± 2.7	33.8	N.D.	10
*P. absinthioides*	P2H1	17.9 ± 10.3	45.0	N.D.	2.5
	P2H4	5.3 ± 1.9	47.1	N.D.	2.5
	P2H35	15.2 ± 2.0	44.4	N.D.	2.5
	P2H40	9.3 ± 2.2	50.7	N.D.	2.5
	P2H43	11.8 ± 0.3	7.9	N.D.	2.5
	P2H45	16.9 ± 4.9	45.5	N.D.	2.5
	**P2H47**	**27.7 ± 9.4**	**41.7**	**N.D.**	**10**
	P2R21	7.9 ± 1.1	5.6	N.D.	2.5
	P2R22	9.4 ± 1.3	8.5	N.D.	2.5
	P2R24	5.8 ± 0.6	52.1	N.D.	2.5
	P2R32	9.7 ± 0.9	36.5	N.D.	5
	P2R43	8.5 ± 2.5	8.0	N.D.	10
*G. mucronata*	NH1B	10.4 ± 3.7	5.2	N.D.	10
	NH4B	20.0 ± 10.6	4.0	N.D.	5
	NH6B	7.9 ± 2.9	5.9	N.D.	5
	NH7B	21.3 ± 6.7	0.0	N.D.	7.5
	NR3B	8.4 ± 1.3	0.1	0.1	5
	NR10B	7.3 ± 1.0	0.0	N.D.	5
	NH3A	6.5 ± 0.8	0.0	N.D.	5
	NR1A	8.7 ± 1.6	0.1	0.2	5
*H. pilosella*	EH8B	7.4 ± 0.4	1.8	N.D.	10
	EH9B	6.6 ± 0.4	0.0	N.D.	10
	EH12B	25.8 ± 7.2	0.0	N.D.	10
	EH13B	4.6 ± 0.9	0.0	N.D.	10
	ER1B	15.7 ± 2.6	9.6	0.2	7.5

*ACC* 1-aminocyclopropane-1-carboxylic acid deaminase, *IAA* indole acetic acid, *N.D.* not detected.

^†^The values represent the means ± standard deviations from *n* = 4.

^‡^The values represent the media of two technical measurements of the composite samples by HPLC.

*Isolates in bold were further used for effects on wheat plants.

Partial sequencing analysis of the 16S rRNA gene indicated that the 42 endophytic strains with assayed PGP traits belonged to the genera *Bacillus* (23 strains), *Serratia* (8), *Staphylococcu*s (4), *Pantoea* (2), *Acinetobacter* (1), *Variovorax* (1), *Curtobacterium* (1), *Pseudomonas* (1) and *Trichococcus* (1) (Table S1). Among them, the phosphobacteria *Variovorax* sp. P1R9, *Staphylococcus* sp. P1R11, *Bacillus* sp. P1R13, *Bacillus* sp. P1R34, and *Curtobacterium* sp. P2H47 were selected for seed inoculation assays due to their higher values of halotolerance (10% NaCl), ACC deaminase activity and auxin production (Table 2).

### Effects on the germination of salt-exposed wheat seedlings

The effects of the five most promising PGP strains on the germination of salt-exposed (0.15 and 0.25 M NaCl) wheat seeds were quantified by the germination index (*GI*) (Eq. 3). Isolates were inoculated as single strains, dual consortia (consortia 1–3 and 5–7) or triple consortia (consortia 4 & 8). Single-strain application generally led to a lower *GI* of 0.15 M salt-treated seeds (*GI* = 68–163%) than salt-free controls (*GI* = 205–405%) (Table 3). Values of 80% < GI < 50% and GI < 50% indicated intermediate and high salt stress of the seeds, respectively. Only strains *Variovorax* sp. P1R9 (*GI* = 132%) and *Curtobacterium* sp. P2H47 (GI = 60%) were able to alleviate the effects of 0.25 M NaCl on seed germination.

**Table 3:** Effect of putative endophytic isolates and consortia on germination of wheat seeds as quantified by the percentage (%) of seed germination and the germination index (*GI*).

	NaCl (M)	Fraction of germinated seeds (%)	*GI* (%)	Salt-induced stress^a^
Single strain				
*Variovorax* sp. P1R9	0	90	293 ± 97	None
	0.15	81	163 ± 12	None
	0.25	78	132 ± 45	None
*Staphylococcus* sp. P1R11	0	98	205 ± 65	None
	0.15	85	121 ± 27	None
	0.25	55	32 ± 35	High
*Bacillus* sp. P1R13	0	90	241 ± 67	None
	0.15	71	112 ± 9	None
	0.25	55	40 ± 12	High
*Bacillus* sp. P1R34	0	100	336 ± 129	None
	0.15	59	68 ± 35	Intermediate
	0.25	55	57 ± 14	Intermediate
*Curtobacterium* sp. P2H47	0	104	405 ± 187	None
	0.15	81	123 ± 42	None
	0.25	72	60 ± 37	Intermediate
Consortia (1 to 4)				
Consortium 1				
*Variovorax* sp. P1R9 *Staphylococcus* sp. P1R11	0	76	216 ± 36	None
	0.15	79	163 ± 27	None
	0.25	66	91 ± 20	None
Consortium 2				
*Variovorax* sp. P1R9 *Curtobacterium* sp. P2H47	0	82	213 ± 33	None
	0.15	97	190 ± 18	None
	0.25	87	124 ± 16	None
Consortium 3				
*Staphylococcus* sp. P1R11 *Curtobacterium* sp. P2H47	0	66	110 ± 24	None
	0.15	71	90 ± 18	None
	0.25	68	63 ± 13	Intermediate
Consortium 4 (triple)				
*Variovorax* sp. P1R9	0	92	228 ± 25	None
*Staphylococcus* sp. P1R11	0.15	90	167 ± 20	None
*Curtobacterium* sp. P2H47	0.25	74	97 ± 18	None

^a^GI % > 80% = none or absence of salt toxicity; 50 < GI% < 80 = intermediate salt toxicity; GI % < 50 = high salt toxicity.

Applying dual (consortia 1–3) or triple (Consortium 4) consortia to wheat seeds increased *GI* up to elevated salt concentrations (90–190% at 0.15 M NaCl; and 91–124% at 0.25 M NaCl). Inclusion of *Variovorax* sp. strain P1R9 in dual or triple consortia thereby resulted in a consistently higher *GI* than in the absence of the strain, suggesting synergistic salt stress-alleviating effects due to the presence of strain P1R9. Therefore, small differences between dual and triple consortia were found with *GI* = 163–167% (0.15 M NaCl) and 91–97% (0.25 M NaCl) compared to salt-free controls (*GI* = 213–228%).

The potential PGP effect of *Variovorax* sp. strain P1R9 in consortia inoculation was further tested by coinoculation of strain P1R9 with the rhizobacteria *Klebsiella* sp. 8LJA and/or *Klebsiella* sp. 27IJA (Table 4). While single-strain application of *Klebsiella* sp. 8LJA or *Klebsiella* sp. 27IJA exhibited no or intermediate alleviation of salt stress only (*GI* = 37–67%, Table 4), coinoculation with *Variovorax* sp. P1R9 led to *GI* = 86% (0.25 M NaCl) and 198% (0.15 M NaCl) and, hence, high salt stress-alleviating effects (Table 4).

**Table 4:** Effect of the rhizobacteria *Klebsiella* sp. strain 8LJA and strain 27IJA and their consortia with *Variovorax* sp. strain P1R9 on the germination of wheat seeds as quantified by the % of seed germination and the germination index (*GI*).

	NaCl (M)	Relative seed germination (%)	*GI*^a^ (%)	Salt-induced stress^a^
Single strain				
*Klebsiella* sp. 8LJA	0	100	215 ± 31	None
	0.15	90	67 ± 17	Intermediate
	0.25	96	37 ± 24	High
*Klebsiella* sp. 27IJA	0	100	216 ± 51	None
	0.15	90	54 ± 13	Intermediate
	0.25	100	37 ± 21	High
Consortia (5 to 8)				
Consortium 5				
*Klebsiella* sp. 8LJA + *Variovorax* sp. P1R9	0	113	332 ± 74	None
	0.15	106	161.3 ± 59	None
	0.25	103	81 ± 35	None
Consortium 6				
*Klebsiella* sp. 27IJA + *Variovorax* sp. P1R9	0	86	264 ± 40	None
	0.15	93	120 ± 37	None
	0.25	96	62 ± 45	Intermediate
Consortium 7				
*Klebsiella* sp. 8LJA + *Klebsiella* sp. 27IJA	0	83	146 ± 19	None
	0.15	103	63 ± 38	Intermediate
	0.25	80	23 ± 16	High
Consortium 8 (triple)				
*Klebsiella* sp. 8LJA	0	100	342 ± 84	None
*Klebsiella* sp. 27IJA	0.15	116	198 ± 65	None
*Variovorax* sp. P1R9	0.25	86	86 ± 53	None

^a^*GI* > 80% = none or absence of salt stress; 50 < *GI* < 80 = intermediate salt stress; *GI* < 50 = high salt stress.

### Effects on physiological and biochemical responses of salt-exposed wheat plants

**Figure 1:**
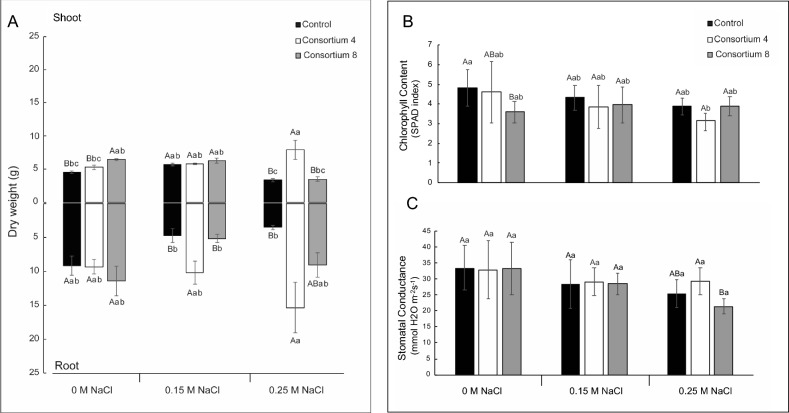
Effect of salt-induced stress on the biomass (**A**), chlorophyll content (**B**) and stomatal conductance (**C**) of wheat seedlings inoculated with *Variovorax* sp. strain P1R9 as part of the root endosphere (Consortium 4) and a rhizosphere consortium (Consortium 8) (Tables 1, 2). Bars represent mean values, while error bars represent standard deviation. Upper–case letters denote statistical differentiation within each salt (NaCl) treatment. Lower–case letters denote statistical differentiation between all samples (*P* ≤ 0.05; Tukey’s test).

Greenhouse experiments were conducted to further test the effects of triple consortia 4 and 8 containing *Variovorax* sp. P1R9 on wheat seedlings by analyzing shoot and root biomass, leaf chlorophyll contents, stomatal conductance, lipid peroxidation (TBARS) and antioxidant enzymes (SOD and CAT) under salt stress conditions (Fig. 1, Table 4). The analyses showed that both consortia generally had beneficial impacts on the growth and well-being of plants exposed to salt stress.

#### Plant biomass

Uninoculated wheat plants showed significantly reduced (P < 0.05) root and shoot biomass upon exposure to 0.25 M NaCl salt compared with the salt-free control (Fig. 1A). Consortium 4 significantly increased (P < 0.05) shoot biomass (7.9 g) relative to uninoculated controls at 0.25 M NaCl (3.3 g). Similarly, Consortium 4 further caused higher root biomass at 0.25 M NaCl (15.3 g) relative to uninoculated controls at 0.25 M NaCl (3.6 g) and with the salt-free control (9.3 g). Compared with uninoculated controls (3.6 g), Consortium 8 also significantly promoted (P < 0.05) root growth at 0.25 M NaCl (9.1 g) but did not lead to beneficial effects on the shoots of wheat exposed to salt.

#### SPAD chlorophyll content

Leaves of uninoculated wheat plants exposed to salt showed lower, but not significant (P < 0.05), SPAD chlorophyll contents (4.3 and 3.8 SPAD units) relative to NaCl-free controls (4.8 SPAD units). Plants inoculated with either of the two triple consortia had similar SPAD indices compared to uninoculated control plants (Fig. 1B).

#### Stomatal conductance

Exposure of seedlings to 0.25 M NaCl resulted in an ~ 20% decrease in stomatal conductance in wheat leaves (Fig. 1C), which could be counteracted by the presence of Consortium 4 (but not of Consortium 8). However, both triple consortia had no significant (P < 0.05) beneficial impact on stomatal conductance at 0.15 M NaCl.

#### Lipid peroxidation

The presence of either of the triple consortia led to significantly (P < 0.05) lower TBARS levels (~ 9–17 nmol MDA g^−1^ FW) than that in uninoculated controls (~ 22 nmol MDA g^−1^ FW), indicating a stress-reducing effect of the inocula (Table 5).

**Table 5:** Effect of NaCl stress on superoxide dismutase (SOD), catalase (CAT) and thiobarbituric acid reactive substances (TBARS) activity of wheat seedlings inoculated with selected triple consortia 4 and 8.

Inoculation	NaCl (M)	SOD (U mg protein^−1^)	CAT (nmol min^−1^ mg protein^−1^)	TBARS (nmol MDA g^−1^ FW)
Control	0	61.4 ± 2.7 cd	30.4 ± 4.7 c	22.0 ± 0.8 a
	0.15	106.8 ± 3.1 ab	72.5 ± 8.2 c	21.5 ± 0.2 a
	0.25	25.1 ± 6.5 d	142.2 ± 6.9 bc	22.4 ± 1.0 a
Consortium 4	0	73.6 ± 1.6 bc	38.2 ± 4.8 c	14.1 ± 2.2 bc
	0.15	142.9 ± 5.3 a	93.1 ± 17.2 c	9.4 ± 1.4 cd
	0.25	55.4 ± 4.9 cd	300.4 ± 40.7 a	14.3 ± 0.3 bc
Consortium 8	0	67.3 ± 6.7 bcd	96.0 ± 28.3 c	7.7 ± 0.1 d
	0.15	36.6 ± 12.3 cd	149.3 ± 20.9 bc	12.7 ± 0.5 bcd
	0.25	28.3 ± 4.8 d	246.8 ± 7.5 ab	17.3 ± 0.2 ab

Different letters denote significant differences (P ≤ 0.05; Tukey’s test) among treatments.

#### Superoxide dismutase

Exposure of uninoculated seedlings to salt (0.15 M NaCl) led to twofold higher SOD activity than that in salt-free controls (Table 5). Plants inoculated with Consortium 4 showed higher SOD activity (33% and 121%) than uninoculated control plants at 0.15 M and 0.25 M NaCl, respectively. In contrast, similar SOD activity as that in uninoculated controls was observed in the presence of Consortium 8 at both salt levels. This indicates that bacterial inoculation induced the regulation of antioxidative enzymes against salt stress.

#### Catalase

The catalase activity of salt-exposed plant controls was higher (72–300 nmol min^−1^ mg protein^−1^) than that of salt-free controls (~ 30 nmol min^−1^ mg protein^−1^; Table 5), while the increase in plants inoculated with the triple consortia was approximately three to eightfold. Seedlings exposed to 0.25 M NaCl had CAT activities of ~ 300 nmol min^−1^ mg protein^−1^ (Consortium 4) and ~ 247 nmol min^−1^ mg protein^−1^ (Consortium 8), which were higher than those in uninoculated plants (~ 142 nmol min^−1^ mg protein^−1^).

### Effects on the abundance and alpha- and beta-diversity of salt-exposed wheat rhizobacterial communities

**Figure 2:**
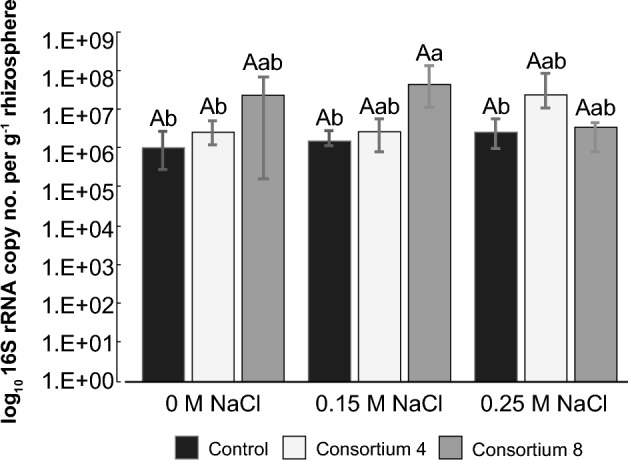
Effect of inoculation on wheat seedling rhizobacterial community size (measured as 16S rRNA) inoculated with *Variovorax* sp. strain P1R9 as part of the root endosphere (Consortium 4) and a rhizosphere consortium (Consortium 8) (Tables 1, 2). Bars represent mean values, while error bars represent standard deviation. Upper-case letters denote statistical differentiation within each salt (NaCl) treatment. Lower-case letters denote statistical differentiation between all samples (*P* ≤ 0.05; Tukey’s test).

In salt-free controls (Fig. 2), the qPCR analyses revealed higher (but not significant, P ≤ 0.05) bacterial gene abundances in rhizosphere samples from plants inoculated with consortia 4 or 8 (~ 4 × 10^7^ 16S rRNA genes g^−1^ sample) compared with uninoculated plants (1.6 × 10^6^ 16S rRNA genes g^−1^ sample). At 0.15 M NaCl, significantly higher (P ≤ 0.05) abundances were observed in the rhizosphere of plants inoculated with Consortium 8 (7.4 × 10^7^ copies of 16S rRNA genes g^−1^ sample) than in the rhizosphere of plants inoculated with Consortium 4 (4.2 × 10^6^ copies of 16S rRNA genes g^−1^ sample) or that of uninoculated plants (2.4 × 10^6^ copies of 16S rRNA genes g^−1^ sample). In contrast, at 0.25 M NaCl, no significant differences were observed among the treatments (Fig. 2).

**Figure 3:**
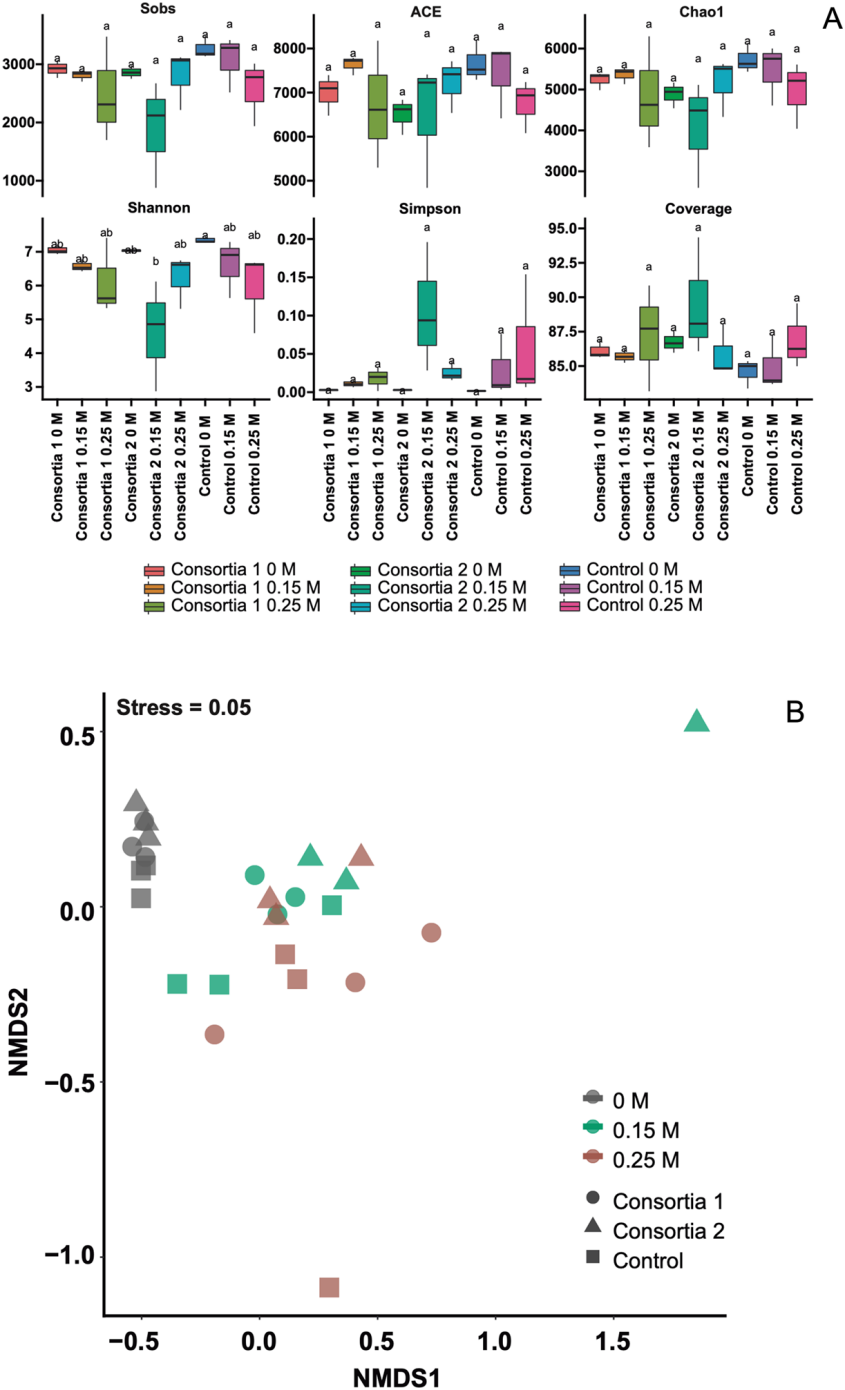
Alpha and beta diversity indices of rhizobacterial communities associated with wheat seedlings inoculated with *Variovorax* sp. strain P1R9 as part of the root endosphere (Consortium 4) and a rhizosphere consortium (Consortium 8) (Tables 1, 2). (**A**) Diversity metrics (ACE, Chao1, Coverage, Shannon, Simpson and Sobs) were calculated using a subset of 25,000 reads per sample. Horizontal bars in the box plots indicate median proportional values. The lower and upper edges of the boxes represent the approximate 1st and 3rd quartiles, respectively. Different lower-case letters indicate significant differences (*P* < 0.05) among treatments. (**B**) Nonmetric multidimensional scaling (NMDS) based on Bray–Curtis dissimilarity showing the structures of the rhizobacterial community from salt-stressed wheat plant samples.

Sequencing proposed similar abundance–based coverage estimates (ACE); however, it revealed no differences between controls and salt-exposed plants regardless of treatment. ACE values ranged from 4160 to 4976 (0.25 M NaCl), 4314 to 4841 (0.15 M NaCl) and 4306 to 4565 (salt-free controls) (Fig. 3A). The Chao1 abundance index, as an indicator of richness changes across samples, was similar for all treatments. Likewise, sequencing coverage values (at 97% similarity) showed no differences.

The Shannon index, a measure of alpha diversity, did not show significant differences (*P* < 0.05) among treatments, except in plants treated with Consortium 8 at 0.15 M NaCl (Fig. 3A). Similarly, no differences in the Simpson diversity index were found among any of the plant treatments. Finally, nonmetric multidimensional scaling (NMDS) analysis revealed a highly heterogeneous composition of rhizosphere bacterial communities of wheat seedlings (Fig. 3B). Analysis of similarity (ANOSIM) thereby confirmed a separation between salt-free controls and salt treatments, where NaCl explained 51.5% of bacterial community variations (P < 0.001). However, no clustering in response to inoculated and control treatments was found (Fig. 3B).

## Discussion

In this study, we isolated and characterized putative endophytic phosphobacteria with multiple PGPB traits associated with the roots of plants growing in the arid Atacama Desert and semiarid Patagonian regions in Chile. Isolates with the most promising PGP traits were tested as single strains, in consortia, and in combination with previously isolated PGP rhizosphere bacteria for their effects on the germination of wheat plants exposed to varying salt stress conditions in the laboratory. Of the initial 68 endophytic phosphobacteria selected and evaluated, 35 showed combined halotolerance (Table 2) with potential PGP traits (Table 1), confirming previous reports on combined halotolerance and ACC deaminase activity in endophytes from extreme habitats^14,22^ and the potential of endophytic bacteria as beneficial plant inoculants^9^. ACC deaminase activity may play a crucial role in plant stress tolerance by lowering ethylene levels through ACC deamination^23^. It has been described that environmental bacteria might express wide variability in ACC-degrading activity^24^. Our reported ACC deaminase activity was higher (~ 5–28 µM α-ketobutyric acid mg^−1^ h^−1^) than that in avocado plant endophytes (~ 0.2–3.6 µM α-ketobutyric acid mg^−1^ h^−1^)^18^ but was lower than that in plant isolates derived from the Atacama Desert or Antarctica (~ 13–40 mmol α-ketobutyric acid mg protein^−1^ h^−1^)^12,14^. The studied endophytes also produced auxin via the tryptophan-based pathway (Table 2), while only four isolates produced IAA at elevated concentrations in the range of 0.2–1.3 mg L^−1^ (Table 2), i.e., values similar to those in endophytes of *Thymus vulgaris*^25^ but lower than those in endophytes of rice seedling roots (34.2 mg L^−1^)^26^.

Hypothesizing that endophyte bacteria of wild plants in extreme environments possess PGP effects on, e.g., the germination of salt-exposed weed seedlings, we selected five highly halotolerant strains (10% NaCl) with high ACC deaminase activity for further testing. 16S rRNA gene sequencing characterized them as species of *Variovorax, Staphylococcus, Bacillus*, and *Curtobacterium* (Table 3), all genera that have been previously described as symbionts of the rhizo- and endospheres of wild plants^27,28^. All five strains were tested individually, and the three strains with the highest *GI* at 0.15 M NaCl were used in dual and triple consortia. Although fractions of seeds germinating under salt exposure were lower than in salt-free controls, the presence of *Variovorax* sp. P1R9, *Staphylococcus* sp. P1R11, *Bacillus* sp. P1R13, or *Curtobacterium* sp. P2H47 led to increased *GI* (112–163%; Table 3) and hence to positive effects on seed germination at 0.15 M NaCl.

Interestingly, *Variovorax* sp. P1R9 thereby showed the highest *GI* at both salt levels (Table 3). This finding supports previous descriptions of PGP by *Variovorax* on PGP^29^ and its association with plant species, such as *Beta vulgaris*^30^, *Gossypium hirsutum*^31^, and *Solanum lycopersicum*^32^. Comparative genomic analysis suggests that members of the genus *Variovorax* can use a wide range of organic compounds, including toxic compounds such as 4-hydroxybenzoate and BTEX^33,34^. Such metabolic versatility may foster its symbiotic lifestyle with diverse plant taxa and allow it to cope with multiple stress conditions. *Variovorax* sp. has been used to study microbe–microbe and microbe–plant interactions in model systems^35^. For instance, Chen et al.^36^ found that the *Variovorax paradoxus* 5C-2 strain with ACC deaminase activity enhanced leaf growth and flowering of *Arabidopsis thaliana*. Moreover, Bessadok et al.^37^ reported that endophytic *Variovorax* sp. CT7.15 improved the in vitro seed germination, nodulation and growth of *Calicotome villosa* plants. They also found more plant growth promotion (e.g., phosphate solubilization and ACC deaminase activity) and nodulation when plants were coinoculated with wild-type rhizobia in arid Tunisian soils. In our study, we also assessed the possible synergetic effects of dual consortia of *Variovorax* sp. P1R9 with either *Staphylococcus* sp. P1R11 or *Curtobacterium* sp. P2H47 (consortia 1–3) as well as a triple consortium of all three strains (Consortium 4) on wheat seed germination (Table 3). We found that the consortia containing *Variovorax* sp. P1R9 consistently exhibited the highest *GI* values, suggesting that *Variovorax* sp. P1R9 had a beneficial effect on the germination of seeds exposed to salt stress (Table 3). However, competence or synergisms of *Variovorax* sp. P1R9 with other members of the consortia need to be demonstrated in further studies.

Given the beneficial role of *Variovorax* sp. P1R9 in endophytic consortia, we also tested its possible synergetic PGP effects in consortia with two known stress-alleviating PGP *Klebsiella* sp. strains isolated from a prior Atacama Desert altiplano^14^. Single or joint application (Consortium 7) of *Klebsiella* sp. strains resulted in intermediate to low salt stress-alleviating effects, and the combination with *Variovorax* sp. P1R9 strains in dual (consortia 5 & 6) or triple consortia (Consortium 8) led to a higher *GI* (Table 3) than the presence of only *Klebsiella* sp. Although the mechanism of such synergistic effects remains unclear, it may be associated with the ability of *Variovorax* strains to convert various substrates produced by other biota (e.g., acyl homoserine lactones (AHLs) and alkyl/aryl-sulfonates) into cell biomass^34^. This metabolic capacity also suggests that *Variovorax* may also exert significant roles in plant nutrient acquisition and natural biogeochemical cycling of soil nutrients. For instance, studies have shown that *V. paradoxus* 5C-2 led to increased soil nitrogen contents and increased plant stress, lowering abscisic acid concentrations in *Pisum sativa* plants^38,39^. Other studies have also shown that the production of ACC deaminase and IAA by *Variovorax* strains improve plant salt stress tolerance^36,40,41^. PGP effects of *Variovorax* sp. HRRK 170 were also linked to an optimal inoculum cell density and subsequent colonization of roots of Chinese cabbage and green pepper^30^.

Hence, we further studied the effects of the *Variovorax* sp. strain P1R9 within consortia 4 and 8 on the physiological and biochemical responses of salt-exposed wheat plants in greenhouse experiments. Endophytic Consortium 4 increased the biomass of shoots and roots in seedlings exposed to 0.25 M NaCl, while mixed Consortium 8 only had stress-alleviating effects on root biomass (Fig. 1). This is in agreement with the results of Barra et al.^18^, which showed stronger PGP effects of endophytic than of rhizosphere bacterial consortia upon inoculation of salt-exposed avocado plants. The close innate interactions of endophytes with host plants seem to be a driver for the beneficial inoculation of plants exposed to salt stress. Endophytic bacteria have been reported to be more efficient in inducing antioxidant activity responses against stresses than rhizosphere microbes^14,22^.

Higher stomatal conductance in plants inoculated with Consortium 4 would support this hypothesis. Additionally, Flores-Duarte et al.^28^ reported that the endophytic *Variovorax gossypii* JM-310T enhanced the nodulation and chlorophyll and nitrogen contents of *Medicago sativa* plants, ameliorating the physiological status of plants in response to moderate and high levels of contamination. They also found improved plant growth and nodulation after inoculation with *V. gossypii* JM-310T, *Ensifer medicae* MA11 and *V. paradoxus* S110T.

The possible beneficial effects of endophytes were further followed by analyzing SOD, CAT and TBARS activity, i.e., antioxidant plant responses to salt stress. PGPB have been reported to enhance enzymatic antioxidant reactions in plants^1,8,18,28^, thereby contributing to stress mitigation. As shown in Table 5, TBARS levels were similar in uninoculated plants independent of the salt level. However, inoculation significantly decreased TBARS levels in controls and salt-exposed plants, with the highest decrease occurring in the presence of Consortium 4 at 0.15 M NaCl. This also confirms a previous observation that PGPB decrease TBARS levels in plant tissues^18^. Chandra et al.^42^ further showed that inoculation of wheat plants with *Variovorax paradoxus* RAA3, *Pseudomonas palleroniana* DPB16 or *Pseudomonas* sp. UW4 decreased TBARS and H_2_O_2_ contents and increased enzyme activities (SOD and CAT, among others) of wheat plants under field conditions with variable water supply. Similar to our results (Table 5), these authors found that the presence of a *Variovorax* strain (*V. paradoxus* RAA3) led to the highest increase in antioxidant enzymes under drought stress conditions compared to controls.

On the other hand, analysis of the diversity of the wheat seedling rhizobiome revealed clear differences between salt-exposed and control consortia yet minor differences upon inoculation with both consortia in both salt-free controls and salt-exposed plants (Fig. 3). Salt-induced changes in rhizosphere microbial community structure have also been reported by Xu et al.^43^.

## Conclusions

This study demonstrated the association of wild plants (*Distichlis spicata*, *Pluchea absinthoides*, *Gaultheria mucronata* and *Hieracium pilosella*) grown in extreme Chilean biomes with potential PGP endophytic bacteria. Forty-two isolates were characterized as nonredundant halotolerant auxin- and ACC deaminase-producing bacteria of the genera *Variovorax*, *Bacillus*, *Staphylococcus* and *Curtobacterium*, with strain *Variovorax* sp. P1R9 showing the best mitigation of salt-induced toxicity on wheat (*T. aestivum* var. Fritz) seed germination. However, the highest seed germination rates under salt stress were observed when strain P1R9 was coinoculated in a triple consortium with the PGP endophytes *Staphylococcus* sp. P1R11 and *Curtobacterium* sp. P2H47 (Consortium 4) and PGP rhizobacteria *Klebsiella* sp. 8LJA or *Klebsiella* sp. 27IJA (Consortium 8). The endophyte consortium also showed the best PGP effects on the physiological and biochemical responses of wheat seedlings when exposed to salt stress. Although the underlying mechanisms still need to be clarified, our data suggest that the application of *Variovorax* sp. strain P1R9 as a part of bacterial consortia may counteract the negative effects of soil salinity on wheat crops. Finally, future agricultural techniques for the promotion of plant growth should consider microbial interactions in plant metaorganisms.

## Materials and methods

### Plant collection and isolation of culturable plant endophytes

Three wild specimens of each extremophyte species, *Distichlis spicata* (Poaceae) and *Pluchea absinthoides* (Asteraceae) from the Atacama Desert (AD; 23° 1ʹ 59ʹʹ S, 68° 11ʹ 59ʹʹ W) and *Gaultheria mucronata* (Ericaceae) and *Hieracium pilosella* (Asteraceae) from Chilean Patagonia (PAT; 53° 28ʹ 0ʹʹ S, 71° 0ʹ 59ʹʹ W), were collected from public lands according to the Chilean legislation. The specimens were randomly sampled from a 10 m transect by using a clean spade to remove intact roots from the soil, placed in plastic bags and then immediately transported on ice to the Applied Microbial Ecology Laboratory (EMALAB) at Universidad de La Frontera (Temuco, Chile) for microbiological analyses. Culturable endophytic bacteria were isolated as described previously by Rilling et al.^7^. Briefly, pieces of roots and leaves were separated and surface sterilized by repeated immersion in 70% (v/v) ethanol for 3 min, followed by 2.5% (v/v) sodium hypochlorite (NaOCl) for 5 min. After disinfection, the root and leaf samples were exhaustively rinsed with sterile distilled water (SDW), and tissue portions (2 g) were dissected and aseptically macerated with a sterile mortar and pestle. Root and leaf tissue homogenates were placed in sterile plastic tubes, mixed into 50 mL of sterile saline solution (0.85% NaCl), serially diluted, and plated onto 1.5% agar plates containing tenfold diluted Luria–Bertani (LB) medium and NM-1 minimal medium^7^. Both culture media were amended with 10 μg mL^−1^ cycloheximide to prevent fungal growth.

Colonies grown on agar plates were counted after 4 days of incubation at 30 °C. Colonies with different phenotypes (color, sizes, shapes, brightness, and elevation) were then randomly chosen and transferred to and grown in fresh culture media. Three hundred seventy-six isolates were purified on agar plates by streaking. Isolates were stored at − 80 °C in LB:glycerol (7:3) and used for further assays.

### Screening for PGP activities

Cereal production in Chilean relays is implemented on ash-derived volcanic soils (Andisol), which are recognized by a high content of total phosphorus (P) and organic matter, and P-releasing ability is the main trait in the study and selection of potential PGPB, also known as phosphobacteria^44^. Thus, all isolates were first tested for their ability to use insoluble forms of inorganic (Pi) and organic (Po) phosphorus as described by Jorquera et al.^43^. All isolates were grown at 30 °C for 2–4 days on National Botanical Research Institute’s phosphate growth medium (NBRIP) and Pikovskaya’s medium (PVK) agar plates supplemented with insoluble tricalcium phosphate (Ca_3_[PO_4_]_2_) as the sole Pi source. Phytate-screening medium (PSM) supplemented with insoluble phytic acid dodecasodium salt hydrate (C_6_H_18_O_24_P_6_ × 12Na × xH_2_O) as the sole Po source was also used. Isolates that grew with clear halos on agar as a result of bacterial growth were considered Pi- and Po-utilizers and selected for additional testing of putative ACC deaminase activity and the production of tryptophan–dependent auxins^45^. Isolates were grown in 5 mL DF minimal medium supplemented with (NH_4_)_2_SO_4_ as the sole source of nitrogen and incubated at 30 °C for 2 days^46^. Bacterial cells were collected by centrifugation (3000×*g* for 5 min) and exhaustively rinsed with 1 mL of sterile salt solution, and then 0.1 mL aliquots were transferred to fresh DF broth supplemented with 3 mM 1-aminocyclopropane-1-carboxylic acid (ACC) as the sole source of nitrogen (N). Isolates growing in ACC-supplemented DF broth were considered ACC deaminase positive.

The production of tryptophan-dependent auxins was colorimetrically determined using Salkowski’s reagent (150 mL of concentrated H_2_SO_4_, 250 mL of distilled H_2_O, 7.5 mL of 0.5 M FeCl_3_ × 6H_2_O) as described by Acuña et al.^45^. Each isolate was grown in LB broth supplemented with 5 mM L^−1^ tryptophan. After incubation at 30 °C for 36 h, bacterial cells were centrifuged (10,000×*g* for 10 min), and the supernatants were vigorously mixed 1:2 with Salkowski’s reagent. Supernatants showing a red coloration after incubation at room temperature for 30 min were considered positive for tryptophan-dependent auxin production. For dereplication of isolates, enterobacterial repetitive intergenic consensus polymerase chain reaction (ERIC-PCR) was performed with the 1R (5ʹ–ATG TAA GCT CCT GGG GAT TCA C–3ʹ) and 2F (5ʹ–AAG TAA GTG ACT GGG GTG AGC G–3ʹ) primer sets as described by Cid et al.^47^.

### Quantification of PGP activities and identification of putative PGP isolates

The ACC deaminase activity of the putative PGP endophytes was quantified by α-ketobutyrate production that was quantified on a microplate reader at 540 nm using an α-ketobutyrate (≥ 97%) calibration curve (0.1–1.8 µM) according to Penrose and Glick^23^. Tryptophan-dependent auxins were quantified at 280 nm by HPLC using a DAD Shimadzu, LC20AT pump, CTO 20AC furnace, a DAD SPD M20A detector and a C18 reversed-phase column (5 μm, 4.6 × 100 mm^−2^) based on calibration with pure auxin indole acetic acid (IAA) using concentrations ranging from 0 to 75 μg mL^−1^.

Halotolerance of putative PGP endophytes was assayed as described by Barra et al.^18^. Isolates were grown in fresh LB broth and plated onto LB agar plates supplemented with 2.5, 5.0, 7.5 and 10.0% NaCl. Compared with the control (0.5% NaCl), colonies grown and showing the same size in media supplemented with NaCl were considered halotolerant. Halotolerant colonies with PGP activities were characterized by partial sequencing of their 16S rRNA genes. Partial amplification of the 16S rRNA gene was performed by endpoint PCR using the 27f (5ʹ‒AGA GTT TGA TCC TGG CTC AG‒3ʹ) and 1492r (5ʹ‒TAC GGY TAC CTT GTT ACG ACT T‒3ʹ) primer set^46^. The sequences were compared with those present in the GenBank database using the BLASTn tool (https://blast.ncbi.nlm.nih.gov/Blast.cgi).

### Effects on the germination of salt-exposed wheat seeds

A germination toxicity assay was conducted using isolates with high ACC deaminase activity, auxin production and salt tolerance on commercial wheat seeds (*T. aestivum* var. Fritz). Commercial wheat seeds (Fritz variety; Semillas Baer Ltd., Chile; https://semillasbaer.cl/) were purchased, and assays were carried out according to the guidelines and recommendation of the Agricultural Research Institute (INIA, https://www.inia.cl/). The selected bacteria (*Variovorax* sp. P1R9, *Staphylococcus* sp. P1R11, *Bacillus* sp. P1R13, *Bacillus* sp. P1R34, and *Curtobacterium* sp. P2H47) were applied as single strains. Although members of *Staphylococcus* are traditionally associated with public health, *Staphylococcus* sp. P1R11 was included in this study because recent studies have reported nonpathogenic *Staphylococcus* as PGPB, particularly to alleviate salt stress in plants^48,49^). The seeds were surface sterilized by repeated immersion in 70% (v/v) ethanol for 3 min, followed by 5% (v/v) NaOCl for 5 min. The seeds were then exhaustively rinsed with SDW, dried over sterile filter paper on Petri dishes in a laminar flow hood for 2 h (*n* = 30 seeds per plate), wetted with SDW and placed in a plant growth incubation chamber for three days. All treatments were performed in quadruplicate. On Day 3, bacterial cultures were prepared in LB tubes, incubated for 24 h at 30 °C, pelletized via centrifugation (3000×*g* for 3 min), and resuspended in sterile 0 M SDW (control) or 0.15 and 0.25 M NaCl SDW. Then, the filter paper of each plate was irrigated with 10 mL of each suspension and incubated for 7 days at 30 °C. After 10 days, germinated seeds were counted, the radicle mean length was quantified, and stress-induced toxicity was measured via the germination index (*GI*)^50^, as derived from Eqs. (1)–(3), with RSG being the relative seed germination (%) and RG the relative growth (%) as follows:

1 $$RSG (\%)= \frac{number\, of \,seeds\, germinated\, at\, salt \,conditions}{number\, of \,seeds\, germinated\, in\, control} \times 100,$$

2 $$RG (\%)= \frac{mean \,root\, length \,at\, salt \,conditions}{\sqrt{mean\, root\, length\, in\, control} } \times 100,$$

3 $$GI \left(\%\right)= \frac{RSG \times RG}{ 100},$$

where *GI* ≥ 80% indicates the absence of salt stress, 80% < *GI* < 50% intermediate salt stress and *GI* < 50% high salt stress.

The three best-performing strains were selected and tested for effects in consortia application using the following consortia: Consortium 1: *Variovorax* sp. P1R9 + *Staphylococcus* sp. P1R11; Consortium 2: *Variovorax* sp. P1R9 and *Curtobacterium* sp. P2H47; Consortium 3: *Staphylococcus* sp. P1R11 and *Curtobacterium* sp. P2H474; and Consortium 4: *Variovorax* sp. P1R9, *Curtobacterium* sp. P2H47, and *Staphylococcus* sp. P1R11.

In a second set of experiments, the effects of coinoculation of *Variovorax* sp. P1R9 with known halotolerance-inducing rhizosphere bacteria from the Atacama Desert (*Klebsiella* strains 8LJA and 27IJA)^14^ on *GI* were tested as single-strain inoculation and in consortia as follows: Consortium 5: *Variovorax* sp. P1R9 and *Klebsiella* sp. 8LJA; Consortium 6: *Variovorax* sp. P1R9 and *Klebsiella* sp. 27IJA; Consortium 7: *Klebsiella* sp. 8LJA and *Klebsiella* sp. 27IJA; and Consortium 8: *Variovorax* sp. P1R9, *Klebsiella* sp. 8LJA, and *Klebsiella* sp. 27IJA.

### Effects on physiological and biochemical responses of salt-exposed wheat plants

The consortia with the highest salt stress toxicity alleviation effects on wheat seed germination (consortia 4 & 8) were tested for their effects on wheat plants (*T. aestivum* var. Fritz) growing for 45 days under greenhouse conditions. One hundred commercial wheat seeds were sown in plastic pots containing 600 g of 3:1 soil:perlite PP8® and incubated in an open greenhouse during summer. The pots were regularly irrigated with DW. At Days 5, 15, and 30, plants were treated with 100 mL of the corresponding bacterial consortia in a 1:1:1 mixture of the individual strains (~ 1 × 10^8^ cells mL^−1^), and each plant was supplemented with NaCl treatment (0.15 and 0.25 M NaCl). Inoculation by irrigation was chosen because it is the most common strategy used by Chilean farmers to introduce microbial inoculants in Chilean crops. Untreated pots (salt stress and bacteria) served as controls. After 45 days, the chlorophyll content and stomatal conductance were measured in the leaves of three plants per pot by using an MC-100 chlorophyll concentration meter (Apogee®, Instruments, Logan, UT, USA) and an SC-1 porometer (Decagon® Devices, Pullman, WA, USA), respectively. Immediately thereafter, roots and shoots were harvested, carefully washed, and split into two subsamples. One subset was dried (60 °C for 48 h) to determine the dry weight of biomass^18^, while the other fraction was frozen at − 80 °C for later quantification of thiobarbituric acid–reactive substances (TBARS) and superoxide dismutase (SOD) and catalase (CAT) activities^51–53^. TBARS were quantified using 0.03 g of fresh leaves macerated in 500 µL of 0.2% trichloroacetic acid and centrifuged at 10,000×*g* for 5 min (4 °C). The level of lipid peroxide quantified by the formation of malondialdehyde (MDA) was determined by measuring absorbances at 440 nm, 532 nm and 600 nm. In addition, SOD and CAT activities were evaluated using 0.1 g of fresh leaf tissue. Total crude proteins from the samples were extracted using 50 mM potassium phosphate buffer (pH 7.0), incubated for 5 min, and centrifuged at 10,000×*g* for 15 min (4 °C). SOD was quantified using the inhibition of the photochemical reduction of nitro blue tetrazolium by photochemically generated superoxide radicals, whereas the consumption of H_2_O_2_ was used to estimate CAT activity. Protein contents were determined by the Bradford method to normalize antioxidant activities and lipid peroxidation.

Rhizosphere soil (i.e., soil aggregates adhering to the roots) was collected in 50 mL tubes upon vigorous shaking of the roots and then used to determine the abundance and structure of the total bacterial community by quantitative PCR (qPCR) and DNA metabarcoding analysis. The moisture content of the rhizosphere was also determined. Tracking or monitoring of each inoculated bacterial strain in the rhizosphere and endosphere of inoculated plants was not performed because specific molecular markers have not been developed thus far, and it is still one of the main challenges in the use of PGPB^54^.

### Effect on salt-exposed wheat rhizosphere bacterial communities

The total DNA of approximately 0.25 g of rhizosphere soil was extracted using a DNeasy PowerSoil Kit (Qiagen, Inc.). DNA size was determined via dye-based quantitative PCR (qPCR), and the DNA sample concentration was fluorometrically determined and adjusted to a concentration of 20 ng μL^−1^. Total bacterial community qPCR (16S rRNA gene) was performed with the chloroplast–mitochondria–excluding primers 799F (5′–AAC MGG ATT AGA TAC CCK G–3′)–1115R (5′–AGG GTT GCG CTC GTT G–3′)^7^. The reactions were set up with a final volume of 10 µL with Solis Biodyne HotFire FirePol SuperMix 2X (Solis Biodyne, Estonia), with 1 µL (0.4 µM) primer per reaction. All reactions were carried out in triplicate and compared to a standard curve ranging from 3 to 3 × 10^−7^ ng µL^−1^ of the 16S rRNA gene from *E. coli* (GenBank accession no. J01859.1), synthesized by IDT (IDT, USA), and amplified with the samples. The obtained 16S rRNA copy numbers were normalized to 1 g of rhizosphere, using the amount of dry soil sample used for the DNA extraction as caliber.

Bacterial communities from each extract were explored by sequencing with Illumina MiSeq^55^. Briefly, the v4 region of the 16S rRNA gene was amplified with the 341F (5′–CCT ACG GGN GGC WGC AG–3′) and 805R (5′–GAC TAC HVG GGT ATC TAA TCC–3′) primer sets coupled in the 5′-end to overhang sequences (5′–ACA CTC TTT CCC TAC ACG ACG CTC TTC CGA TCT–3′; 5′–GTG ACT GGA GTT CAG ACG TGT GCT CTT CCG ATC T–3′, respectively). Libraries were indexed using Nextera XTv2 indices and paired-end sequenced in an Illumina MiSeq (Illumina Inc.) with the Illumina MiSeq V3 Sequencing kit. The resulting reads were trimmed and processed under SHI7 to keep only high-quality base data (QC > 35)^56,57^. Trimmed sequences were aligned as operational taxonomic units (OTUs) and rarified to 25,000 reads for biodiversity analysis. Both the richness (observed OTUs, abundance-based coverage estimates, Chao1) and diversity (coverage, Shannon, and Simpson indices) of the microbial communities were analyzed using the mothur program and plotted in R with the “*ggplot2*” package (https://www.r-project.org/). Nonmetric multidimensional scaling (NMDS) was used to ordinate the samples. Differences in beta diversity among the communities were evaluated by analysis of similarity (ANOSIM) using Bray‒Curtis dissimilarity matrices^58^.

### Statistical analysis

The data were analyzed by one-way analysis of variance (ANOVA), and comparisons were carried out for each pair with Tukey’s test using SPSS software version 19 (SPSS, Chicago, IL, USA). Two-way ANOVA was performed to determine the effects of two factors (salt and consortium inoculation) on the parameters of plants and the abundance of the total bacterial community. Differences were considered significant when the P value was ≤ 0.05.

### Ethics approval

All sampling procedures and methods involved in this study were revised and approved with certification No. 017_20 (issued on April 1st, 2020) by The Scientific Ethics Committee from Universidad de La Frontera (CEC–UFRO; https://cec.ufro.cl/), which is certified by the Health Ministry of the Chilean Government (MINSAL; https://www.minsal.cl/), according to relevant Chilean and international guidelines and regulations.

### Supplementary Information

Supplementary Table S1.

## Data Availability

The 16S rRNA gene sequences from the isolated strains were deposited in GenBank (https://www.ncbi.nlm.nih.gov/genbank/) under accession numbers ON392108 to ON392149. Raw sequencing data files derived from 16S rRNA gene metabarcoding analysis were deposited in the NCBI Sequence Read Archive (SRA; https://www.ncbi.nlm.nih.gov/sra) under Accession Number PRJNA865302.
