# Supplementary material for: Variovorax sp. strain P1R9 applied individually or as part of bacterial consortia enhances wheat germination under salt stress conditions
Source: Sci Rep. 2024 Jan 24;14:2070. doi: 10.1038/s41598-024-52535-0 (PMC10808091; doi:10.1038/s41598-024-52535-0)
Supplement: Supplementary file 1 — Supplementary Table S1. [file 41598_2024_52535_MOESM1_ESM.docx]

**Supplementary Table S1.** Taxonomic affiliation of selected putative plant growth‒promoting bacteria isolated from plants from Atacama Desert (*D. spicata*  and *P. absinthioides*) and Patagonia (*G. mucronata* and *H. pilosella*).

| **Isolates** | **Taxonomic group** | **Closest relatives or cloned sequences (accession no.)*** | **Identity** | **Accession no.** |
| --- | --- | --- | --- | --- |
| *D. spicata* |  |  |  |  |
| P1H14 | *Proteobacteria; Gammaproteobacteria* | *Acinetobacter albensis* strain ANC 4874 from natural soil (NR_145641) | 99% | ON392108 |
| P1H28 | *Firmicutes; Bacilli* | *Staphylococcus edaphicus* strain CCM 8730 from Antarctic stone (NR_156818) | 99% | ON392109 |
| P1H34 | *Firmicutes; Bacilli* | *Staphylococcus edaphicus* strain CCM 8730 from Antarctic stone (NR_156818) | 100% | ON392110 |
| P1R2 | *Firmicutes; Bacilli* | Plant growth-promoting *Bacillus velezensis* strain CBMB205 (NR_116240) | 100% | ON392111 |
| **P1R9**** | ***Proteobacteria; Betaproteobacteria*** | ***Variovorax soli* strain GH9-3 from greenhouse soil (NR_043811)** | **99%** | **ON392112** |
| **P1R11** | ***Firmicutes; Bacilli*** | ***Staphylococcus edaphicus* strain CCM 8730 from Antarctic stone (NR_156818)** | **99%** | **ON392113** |
| **P1R13** | ***Firmicutes; Bacilli*** | **Plant growth-promoting *Bacillus velezensis* strain FZB42 (NR_075005)** | **100%** | **ON392114** |
| P1R15 | *Firmicutes; Bacilli* | *Bacillus aerius* strain 24K from soil (NR_118439) | 100% | ON392115 |
| P1R16 | *Firmicutes; Bacilli* | *Bacillus stratosphericus* strain 41KF2a from upper atmosphere (NR_042336) | 100% | ON392116 |
| P1R29 | *Firmicutes; Bacilli* | *Bacillus subtilis* strain SBMP4 from internal tissues of fresh orange (NR_118383) | 100% | ON392117 |
| **P1R34** | ***Firmicutes; Bacilli*** | ***Bacillus halotolerans* strain DSM 8802 (NR_115063)** | **92%** | **ON392118** |
| P1R39 | *Firmicutes; Bacilli* | *Bacillus toyonensis* strain BCT-7112 from soil (NR_121761) | 100% | ON392119 |
| P1R41 | *Firmicutes; Bacilli* | *Bacillus toyonensis* strain BCT-7112 from soil (NR_121761) | 100% | ON392120 |
| P1R42 | *Firmicutes; Bacilli* | *Bacillus toyonensis* strain BCT-7112 from soil (NR_121761) | 100% | ON392121 |
| P1R65 | *Firmicutes; Bacilli* | *Bacillus stratosphericus* strain 41KF2a from soil (NR_118441) | 99% | ON392122 |
| P1R73 | *Firmicutes; Bacilli* | *Bacillus toyonensis* strain BCT-7112 from soil (NR_121761) | 100% | ON392123 |
| P1R81 | *Firmicutes; Bacilli* | *Bacillus toyonensis* strain BCT-7112 from soil (NR_121761) | 100% | ON392124 |
| *P. absinthioides* | |  |  |  |
| P2H1 | *Firmicutes; Bacilli* | *Bacillus toyonensis* strain BCT-7112 from soil (NR_121761) | 100% | ON392125 |
| P2H4 | *Firmicutes; Bacilli* | *Bacillus toyonensis* strain BCT-7112 from soil (NR_121761) | 100% | ON392126 |
| P2H35 | *Firmicutes; Bacilli* | *Bacillus toyonensis* strain BCT-7112 from soil (NR_121761) | 100% | ON392127 |
| P2H40 | *Firmicutes; Bacilli* | *Bacillus toyonensis* strain BCT-7112 from soil (NR_121761) | 100% | ON392128 |
| P2H43 | *Firmicutes; Bacilli* | *Bacillus bingmayongensis* strain FJAT*-*13831 from soil (NR_148248.1) | 100% | ON392129 |
| P2H45 | *Firmicutes; Bacilli* | *Bacillus toyonensis* strain BCT-7112 from soil (NR_121761) | 100% | ON392130 |
| **P2H47** | ***Proteobacteria; Gammaproteobacteria*** | ***[Curtobacterium] plantarum* strain CL63 from leaves (NR_104943)** | **100%** | **ON392131** |
| P2R21 | *Proteobacteria; Gammaproteobacteria* | *Pantoea stewartii subsp. indologenes* strain CIP 104006 from leafspot (NR_104928) | 94% | ON392132 |
| P2R22 | *Firmicutes; Bacilli* | *Bacillus toyonensis* strain BCT-7112 from soil (NR_121761) | 100% | ON392133 |
| P2R24 | *Firmicutes; Bacilli* | *Staphylococcus condimenti* strain DSM 11674 from water pond (MF678874) | 100% | ON392134 |
| P2R32 | *Proteobacteria; Gammaproteobacteria* | *Pantoea allii* strain BD-390 from onion plants and seed (NR_115258.1) | 99% | ON392135 |
| P2R43 | *Firmicutes; Bacilli* | *Bacillus toyonensis* strain BCT-7112 from soil (NR_121761) | 100% | ON392136 |
| *G. mucronata* |  |  |  |  |
| NH1B | *Firmicutes; Bacilli* | *Bacillus toyonensis* strain BCT-7112 from soil (NR_121761) | 99% | ON392137 |
| NH4B | *Proteobacteria; Gammaproteobacteria* | *Pseudomonas moraviensis* strain 1B4 from soil (NR_043314) | 94% | ON392138 |
| NH6B | *Firmicutes; Bacilli* | *Trichococcus alkaliphilus* strain B5 from a high-elevation wetland (NR_159288) | 96% | ON392139 |
| NH7B | *Firmicutes; Bacilli* | *Bacillus aerius* strain 24K from soil (NR_118439) | 100% | ON392140 |
| NR3B | *Proteobacteria; Gammaproteobacteria* | Endophytic *Serratia quinivorans* strain LMG 7887 from rice (NR_114575) | 99% | ON392141 |
| NR10B | *Proteobacteria; Gammaproteobacteria* | Endophytic *Serratia quinivorans* strain LMG 7887 from rice (NR_114575) | 99% | ON392142 |
| NH3A | *Proteobacteria; Gammaproteobacteria* | Endophytic *Serratia quinivorans* strain LMG 7887 from rice (NR_114575) | 99% | ON392143 |
| NR1A | *Proteobacteria; Gammaproteobacteria* | Endophytic *Serratia quinivorans* strain LMG 7887 from rice (NR_114575) | 99% | ON392144 |
| *H. pilosella* |  |  |  |  |
| EH8B | *Proteobacteria; Gammaproteobacteria* | Endophytic *Serratia quinivorans* strain LMG 7887 from rice (NR_114575) | 99% | ON392145 |
| EH9B | *Proteobacteria; Gammaproteobacteria* | Endophytic *Serratia quinivorans* strain LMG 7887 from rice (NR_114575) | 99% | ON392146 |
| EH12B | *Firmicutes; Bacilli* | *Bacillus toyonensis* strain BCT-7112 from soil (NR_121761) | 100% | ON392147 |
| EH13B | *Proteobacteria; Gammaproteobacteria* | Endophytic *Serratia quinivorans* strain LMG 7887 from rice (NR_114575) | 99% | ON392148 |
| ER1B | *Proteobacteria; Gammaproteobacteria* | Endophytic *Serratia quinivorans* strain LMG 7887 from rice (NR_114575) | 99% | ON392149 |

* Based on partial sequencing of 16S rRNA gene and comparison with those present in GenBank database from National Center for Biotechnology Information (NCBI) by using BLAST nucleotide tool.

** Isolates in bold were further used for effects on wheat plants.
